# The efficacy of ivabradine in the treatment of acute myocardial infarction: A protocol for systematic review and meta-analysis

**DOI:** 10.1097/MD.0000000000026396

**Published:** 2021-06-25

**Authors:** Yonggeng Zhang, Shu Sun, Song Yi

**Affiliations:** Department of Cardiology, Yichun People's Hospital, Jiangxi, China.

**Keywords:** acute myocardial infarction, heart rate, ivabradine, meta-analysis

## Abstract

**Background:**

Cardiovascular diseases have become a prominent threat to public health and quality of life. In recent years, some studies have reported that ivabradine can improve the cardiac function and prognosis of patients with acute myocardial infarction (AMI). Therefore, we perform a protocol for systematic review and meta-analysis to evaluate the efficacy of ivabradine for treating AMI.

**Methods:**

This protocol of systematic review and meta-analysis has been drafted under the guidance of the preferred reporting items for systematic reviews and meta-analyses protocols. We will search PubMed, Cochrane Library, Embase, Web of Science, and Medline databases for relevant studies. In addition, we will also collect 4 databases of China: China National Knowledge Infrastructure, China Biomedical Literature Database, China Science Journal Database, and Wan-fang Database. Risk of bias will be assessed using the Cochrane Handbook risk of bias assessment tool version (V.5.1.0). We will use STATA 16.0 software (Stata Corporation, College Station, TX) to perform data analysis.

**Results:**

The results of this systematic review and meta-analysis will be published in a peer-reviewed journal.

**Conclusion:**

We hypothesized that ivabradine can reduce the resting heart rate and improve heart function in patients with AMI.

## 1 Introduction

Cardiovascular disease poses tremendous burden on public health, as well as the global economy.^[1]^ According to the World Health Organization, an estimated 17.5 million people died from cardiovascular disease in 2012, accounting for 31% mortality globally with an annual cost of $193.1 billion in health-care management and about $123 billion in productivity loss as a result of premature death.^[2]^ An acute myocardial infarction (AMI) is a leading cause of morbidity and mortality worldwide with known complications including congestive heart failure, functional and structural myocardial abnormalities, reinfarction, and death.^[3–5]^ The major cause of in-hospital acute AMI mortality remains acute heart failure, despite the introduction of modern treatment strategies.^[6]^ An elevated heart rate in patients with heart failure complicating AMI attenuates the decrease in cardiac output but, with impaired left ventricular filling, increases myocardial oxygen consumption and reduces myocardial perfusion time in the ischaemic heart.^[7,8]^ Thus, elevated heart rate at rest represents a significant predictor of all-cause and cardiovascular mortality in the general population and in patients with cardiovascular disease. Thus, controlling the heart rate has garnered increasing attention in the clinical treatment of AMI.

Ivabradine acts as an I_f_-channel inhibitor to the heart, selectively inhibiting the I_f_-ionic current, which controls the spontaneous diastolic depolarization in the sinus node, thereby regulating the heart rate.^[9]^ The cardiac effect of ivabradine are sinus node-specific, and have no influence on the intra-atrial, atrioventricular, or intraventricular stimulus conduction.^[10]^ Myocardial contractility and ventricular repolarization remain unchanged. Several studies have reported that ivabradine may be effective and safety for patients with AMI. However, these studies have been limited in their ability to provide strong evidences, such as small sample size and inconsistent adherence to modern methodological research standards, making it difficult to draw meaningful conclusions. Therefore, we perform a protocol for systematic review and meta-analysis to evaluate the efficacy of ivabradine for treating AMI.

## 2 Methods

### 2.1 Protocol register

This protocol of systematic review and meta-analysis has been drafted under the guidance of the preferred reporting items for systematic reviews and meta-analyses protocols.^[11]^ Moreover, it has been registered on open science framework (Registration number: DOI 10.17605/OSF.IO/UEJ5K).

### 2.2 Ethics

Ethical approval is not required for this study since it relies on secondary data.

### 2.3 Inclusion criteria

(1) Participants: The diagnosis of AMI in all participants met the 2007 American Heart Association/American College of Cardiology diagnostic criteria, regardless of age and sex.
(2) Intervention: The experimental group was treated with ivabradine combined with the standard regime, and the control group was treated with the standard regime, with no restrictions on the dose and course of treatment.
(3) Outcome measures: Heart rate; left ventricular ejection fraction; pro-brain natriuretic peptide; occurrence of adverse reactions.
(4) Study type: randomized controlled trials.

### 2.4 Exclusion criteria

Duplicate studies, experience summaries, case reports, reviews; studies with incomplete information or animal experiments; studies with fewer than 15 cases; the loss rate of the subjects was >20%.

### 2.5 Search strategy

This study will use the PubMed, Cochrane Library, Embase, Web of Science, and Medline databases. In addition, we will also collect 4 databases of China: China National Knowledge Infrastructure, China Biomedical Literature Database, China Science Journal Database, and Wan-fang Database. We will consider articles published between database initiation and June 2021. Two authors will independently draft and carry out the search strategy. In addition, we manually retrieve other resources, including the reference lists of identified publications, conference articles, and gray literature. The following search terms will be used: “acute myocardial infarction,” “cardiovascular stroke,” “heart attack,” “myocardial infarct,” and “ivabradine.”

### 2.6 Data extraction and quality assessment

All articles will be read fully by 2 investigators adhering to predesigned forms that include study design, participant's general information, outcome measures, and results. When relevant data were missing or unclear, we attempted to contact the original author for complete data. Any disagreement regarding data extraction will be resolved by discussion with a third reviewer. Risk of bias will be assessed using the Cochrane Handbook risk of bias assessment tool version (V.5.1.0). These criteria include the following categories: allocation concealment, random sequence generation, blinding of participants and personnel, blinding of outcome measures, incomplete outcome data, selective reporting, and other risk of biases. Each study will be assigned as “unclear risk,” “low risk,” or “high risk” of bias. We will use the Grading of Recommendations Assessment, Development, and Evaluation approach to conduct a quality assessment for each outcome. The quality of evidence will be recorded as “high,” “moderate,” “low,” or “very low.” For articles in which the information is insufficient, we will try to contact the authors via phone or email to obtain complete information.

### 2.7 Statistical analysis

We will use STATA 16.0 software (Stata Corporation, College Station, TX) to perform analysis. For continuous outcomes, mean difference (MD) will be used as a summary statistic if data measured with same scale, otherwise standardized mean difference (SMD) will be used. For dichotomous outcomes, risk ratio (RR) will be used in the meta-analysis. All of these data will be summarized with a 95% confidence interval (CI). Heterogeneity among trials will be identified by the *I*^2^ and Chi-squared test statistics. If the included studies have high heterogeneity (*I*^2^ > 50%), we will use a random-effects model for pooling data across studies. Otherwise, a fixed-effects model will be used. To evaluate publication bias, we will construct a funnel plot using Cochrane software if the number of included studies is sufficient (>10 studies). A symmetrical funnel plot indicates no possibility of publication bias, whereas an asymmetrical funnel plot indicates a high possibility of publication bias. If we identify publication bias through analysis of the funnel plot, we will discuss possible reasons such as small-study effects.

### 2.8 Sensitivity analysis

To confirm the robustness of the review finding, a sensitivity analysis will be conducted after excluding poor quality studies, outliers, and missing values. We will compare original with sensitivity analysis results.

## 3 Discussion

With the development of society, environmental and human lifestyle changes, the incidence of AMI has increased over recent years, even affecting younger generations and becoming one of the critical causes of death and disability.^[12,13]^ As an independent risk factor of cardiovascular diseases, the increase of resting heart rate is positively associated with the mortality of cardiovascular events from AMI, which leads to the increase of myocardial oxygen consumption amount and the decrease in coronary artery perfusion.^[14,15]^ Therefore, active clinical control of the resting heart rate has become one of the most important methods for treating AMI. β-blocker is widely used to slow down the heart rate, however, it is associated with several additional effects such as negative conduction, negative muscle strength, lower blood pressure, which limits its clinical application.^[16,17]^ Ivabradine can specifically reduce the rhythm of sinus nodules and slow the heart rate with no additional effects. Besides, it can more quickly and efficiently control the heart rate. The advent of ivabradine has brought a new hope to patients with AMI. Therefore, it is necessary to formulate this systematic review and meta-analysis to synthesize these accessible clinical evidences, and we hope this systematic review will provide more comprehensive, reliable, and practical evidence for clinical decision-making and further research.

## Author contributions

**Conceptualization:** Shu Sun.

**Data curation:** Shu Sun.

**Formal analysis**: Shu Sun.

**Funding acquisition:** Yonggeng Zhang.

**Investigation:** Song Yi, Shu Sun.

**Methodology:** Song Yi.

**Software:** Shu Sun.

**Writing – original draft:** Yonggeng Zhang.

**Writing – review & editing:** Yonggeng Zhang.
